# A disintegrin and metalloproteinase domain 10 expression inhibition by the small molecules adenosine, cordycepin and N6, N6-dimethyladenosine and immune regulation in malignant cancers

**DOI:** 10.3389/fimmu.2024.1434027

**Published:** 2024-08-15

**Authors:** Wenqian Zhang, Jiewen Fu, Jiaman Du, Xiaoyan Liu, Jingliang Cheng, Chunli Wei, Youhua Xu, Junjiang Fu

**Affiliations:** ^1^ Key Laboratory of Epigenetics and Oncology, The Research Center for Preclinical Medicine, Southwest Medical University, Luzhou, Sichuan, China; ^2^ Department of Rehabilitation Medicine, The Affiliated Hospital of Southwest Medical University, Luzhou, Sichuan, China; ^3^ State Key Laboratory of Quality Research in Chinese Medicine, Faculty of Chinese Medicine, Macau University of Science and Technology, Taipa, Macao SAR, China

**Keywords:** ADAM10, expression, pan-cancer, immunotherapy, adenosine, CD, m^6^_2_A

## Abstract

A disintegrin and metalloproteinase domain 10 (ADAM10), a member of the ADAM family, is a cellular surface protein with potential adhesion and protease/convertase functions. The expression regulations in cancers by natural products [adenosine (AD) and its analogs, cordycepin (CD), and N6, N6-dimethyladenosine (m^6^_2_A)], and immune regulation are unclear. As results, AD, CD, and m^6^_2_A inhibited ADAM10 expression in various cancer cell lines, indicating their roles in anti-cancer agents. Further molecular docking with ADAM10 protein found the binding energies of all docking groups were <-7 kcal/mol for all small-molecules (AD, CD and m^6^_2_A), suggesting very good binding activities. In addition, analysis of the immunomodulatory roles in cancer showed that ADAM10 was negatively correlated with immunomodulatory genes such as CCL27, CCL14, CCL25, CXCR5, HLA-B, HLA-DOB1, LAG3, TNFRSF18, and TNFRSF4 in bladder urothelial carcinoma, thymoma, breast invasive carcinoma, TGCT, kidney renal papillary cell carcinoma, SKCM and thyroid carcinoma, indicating the immune-promoting roles for ADAM10. *LAG3* mRNA levels were reduced by both AD and CD *in vivo*. ADAM10 is also negatively associated with tumor immunosuppression and interrelated with the immune infiltration of tumors. Overall, the present study determined ADAM10 expression by AD, CD and m^6^_2_A, and in AD or CD/ADAM10/LAG3 signaling in cancers, and suggested a potential method for immunotherapy of cancers by targeting ADAM10 using the small molecules AD, CD and m^6^_2_A.

## 1 Introduction

A disintegrin and metalloproteinase domain 10 [(ADAM10; Online Mendelian Inheritance in Man (OMIM): 602192] also referred to as HsT1871, Kuz, EC 3.4.24.81, and CD156C or CDw156. ADAM10 is cytogenetically located at 15q21.3, encodes 748 amino acids, and has a predicted molecular weight of 84.1 kD (NM_001110.4; NP_001101). The matured protein has 535 amino acids with a predicted molecular weight of 59.3 KD. ADAM10 is a member of the ADAM family possessing α-secretase activities (1). In 1995, Wolfsberg et al. (2) identified several proteins as members of the ADAM family, including ADAM10, and these are cell surface proteins with a unique structure and potential adhesion and protease functions. Rosendahl et al. (3) purified ADAM10 as a TNF-processing enzyme, and Lunn et al. (4) reported that ADAM10 had the TNFα convertase activity. ADAM10 mutations have been reported to be associated with eticulate acropigmentation of Kitamura (OMIM: 615537) (5) and Alzheimer’s disease 18 (AD18) (OMIM: 615590) (1), two genetic disorders. ADAM10 cleaves amyloid precursor protein (APP) to contribute to the pathophysiology of Alzheimer’s disease. *ADAM17* is a critical paralogue of the *ADAM10* gene at the structural and functional level.

The protease/convertase ADAM10 cleaves as many as 40 important substrates, such as Notch, APP, E-cadherin, L-selectin, CD40L, EGFR/HER ligands, EGF, Fas ligand, inducible costimulator ligand, programmed death-ligand 1, Nectin-4, transmembrane activator and CAML interactor, and the “stress-related molecules” UL16-binding proteins, major histocompatibility complex (MHC) class I polypeptide-related sequence A, and MHC class I polypeptide-related sequence B, which are involved in a multitude of biological functions such as autoimmunity, apoptosis, inflammation, cell metabolism, cell adhesion, cancer proliferation, and cancer metastasis (6–9). ADAM10 has been demonstrated to cleave ephrin-A5 to contribute to the metastasis of prostate cancer (10). Thus, ADAM10 is a potential therapeutic target for anti-cancer treatments (6, 11).

Earlier studies found that ADAM10 is required for HIV-1 (human immunodeficiency virus type-1) replication in both primary human macrophages and immortalized cells (12), and for nuclear trafficking, suggesting that ADAM10 is a novel therapeutic target for suppressing HIV-1 before nuclear entry (13). It is well known that the crucial event promoting the invasion of genetic material, SARS-CoV-2, into the host cells is the activation of spike protein (S-protein) by host proteases or proteinases (14). Angiotensin converting enzyme 2 (ACE2) was the first identified and important receptor for SARS-CoV-2, and numerous receptors and co-receptors have been identified (15). ADAM10 alone or in combination with ADAM17 promotes SARS-CoV-2 cellular entry through S-protein mediation and ACE2 shedding (16, 17). As a hallmark of COVID-19 pathology, Jocher et al. (16) identified ADAM17 as a facilitator of SARS-CoV-2 invasion, and ADAM10 is a host factor that is required for syncytium formation in lung cells. This lung cell infection is a transmembrane serine protease 2 (TMPRSS2) protease-independent manner (16, 18). ADAM10 thus might be one of the potential targets for the development of antiviral drugs (7, 16, 19–21).

Adenosine (AD) is an endogenous nucleoside that exerts physiological effects on numerous other systems and tissues of the cardiovascular system and body. Cordycepin (CD) from *Cordyceps militaris*, a Chinese herbal medicine and an analog of AD, exhibits a broad spectrum of anti-virial, anti-inflammatory, hepato-protective, anticancer, antidepressant and neuro-protective activity (22–24). CD has been reported to show a strong binding affinity with both the S-protein of SARS-CoV-2 and Mpro proteins (25). CD also inhibited the expression of SARS-CoV-2 receptors, such as FURIN, TMPRSS2/4, and dipeptidyl peptidase 4 on cancer cells in a dose-dependent manner (14, 26–28). N6,N6-dimethyladenosine (m^6^_2_A), another analog of AD, has been reported to be a modified ribonucleoside from *Mycobacterium bovis* Bacille Calmette-Guérin (29). Both CD and m^6^_2_A are analogs of AD.

Nevertheless, regulation of ADAM10 expression by components from Chinese herbal medicines may have potential in cancer progression/tumorigenesis. It is important to forecast the patients with cancer outcomes by components of small molecules inhibition, immune regulation. In the present study, the expression regulation by small molecule (AD, CD and m^6^_2_A) inhibition, immune regulation in malignant cancers was analyzed.

## 2 Materials and methods

### 2.1 Public databases

Reverse transcription-PCR (RT-PCR) primers were designed through Primer 3 (https://bioinfo.ut.ee/primer3–0.4.0/), using ADAM10 cDNA sequences from the National Center for Biotechnology Information (https://www.ncbi.nlm.nih.gov/nuccore/NM_001110.4/).

### 2.2 Reagents

AD with cat. number A6218 and m^6^_2_A with cat. number N879945 were purchased from Shanghai Macklin Biochemical Co., Ltd., whereas CD with cat. number B20196 was obtained from Chengdu Must Bio-Technology Co., Ltd. DMSO (cat.no. D8148) and RPMI 1640 medium (cat. no. C3010–0500) were purchased from Millipore Sigma. The RNA extraction kit (cat. no. DP419) was purchased from TRANGEN. The RT-PCR kit (code number: FSQ-201) was purchased from Toyobo Life Science. DMEM (cat. no. C3113–0500) was purchased from Shanghai VivaCell Biosciences., Ltd., while FBS was purchased from Invigentech. The anti-ADAM10 polyclonal antibody was purchased from Proteintech Group, Inc. (cat. No. 25900–1-AP).

### 2.3 Cell culture

DMEM with 10% FBS together 1% penicillin-streptomycin was used for BT549 cells (from ATCC), while RPMI 1640 medium with 10% FBS together 1% penicillin-streptomycin was used for H1975 and A549 cells (from ATCC). All cell lines were cultured in a 12-well plate in an incubator at 37°C containing 5% CO_2_. When the cell density reached 50–70%, CD, AD or m^6^_2_A was added to each experimental group at different concentrations (0, 10, 20 and 40 μM) for 24 h (30). Subsequently, proteins for western blotting and total RNA for semi-quantitative RT-PCR were extracted.

### 2.4 Western blotting (WB) analysis

Total cellular lysates were lysed using 1 × EBC buffer and used for western blotting (WB) analysis. In detail, proteins were subjected to sodium dodecyl sulphate (SDS) -polyacrylamide gel electrophoresis (PAGE) and transferred onto the polyvinylidene difluoride(PVDF) membrane (pore size 0.2 μm). After blocked with 5% nonfat milk for RT 2h, the membrane was used to incubate with anti-ADAM10 polyclonal antibody (Cat No25900–1-AP; Proteintech, Wuhan, China). Anti-ADAM10 polyclonal antibody was used at a dilution of 1:4,000. β-actin (Cat No6609–1-Ig;Proteintech, Wuhan, China) served as an internal control. Finally, the blots were soaked in horseradish peroxidase luminescent substrate and visualized using a gel imaging system (Bio-Rad Laboratories, Inc.).

Cycloheximide (CHX, 20µg/ml) was used to treat A549 lung cancer cell lines or BT549 breast cancer cell lines with or without indicated AD or CD treatments, and WB was performed.

### 2.5 Quantitative RT-PCR

We used online primer design software Primer 3.0 (https://primer3.ut.ee/) to design primers ADAM10, LAG3, ACTB primers were designed. The designed primer sequence of ADAM10 were: RT-ADAM10–5: 5’-agcaacatctggggacaaac-3’ (forward primer), RT-ADAM10–3: 5’-cccaggtttcagtttgcatt-3’ (reverse primer). The expected product size was 241bp. The sequences of the primers LAG3 were: RT-LAG3–5: 5’-cagagatggcttcaacgtctc-3’ (forward primer), RT-LAG3–3: 5’-ctggctcacatcctctagtcg-3’ (reverse primer). The expected product size was 241bp. The sequences of the primers ACTB were: RT ACTB-5: 5’-CTCTTCCAGCCTTCCTTCCT-3’ (forward primer), RT-ACTB-3:5’-CACCTTCACCGTTCCAGTTT-3’ (reverse primer). The expected product size was 510 bp. PCR amplification system configuration: 2×Tap PCR Master Mix (Lot: Y2120, TIANGEN, Beijing, China): 5μL, ddH2O: 3μL, primer: 1μL, cDNA: 1μL. PCR amplification procedure: pre-denaturation at 95^0^C for 90s, denaturation at 95^0^C for 30s, annealing at 60^0^C for 30s, and extension at 72^0^C for 30s, 23 cycles for ACTB, and 30 cycles for the ADAM10 or LAG3 of the final extension of 72^0^C for 5 minutes, and 16^0^C for 2 minutes. Final used 1.5% agarose gel for assays.

### 2.6 Molecular docking

The 3D crystal structure of ADAM10 protein (PBD ID: 6BDZ) (extracellular domain, positions: 220–654) was obtained in pdb format in RCSB PDB database (http://www.rcsb.org/pdb) (31). The AD, CD, and m^6^_2_A small-molecule structures ligand files were download from the Pub Chem (https://pubchem.ncbi.nlm.nih.gov/), and used OpenBabel3. 1. 1 (32) (wiki/Main_Page) into pdb format and saved. Then, we used AutoDock Tools (https://ccsb.scripps.edu/mgltools/) (33) and processed target proteins and small molecules in pdb format and saved them in pdbqt format. To ensure the accuracy of the docking results, we set the target protein as the receptor and performed dehydrogenation and hydrogenation. The small-molecule (AD, CD, and m^6^_2_A) were set as ligands and hydrogenated, and torsion bonds were detected. Receptor-ligand docking with AutoDock Vina 1.1.2 (34) (https://vina.scripps.edu/) after setting up the active pockets, and use the docking score binding energy (affinity) to evaluate the binding activity of the ligand and the target: the smaller the binding energy, the smaller the binding energy. For the Grid parameters, Grid spacing was set to 0.375 Å (default). Center grid box values were set to x = 31.68, y = 19.05, and z = 45.96. The number of grid size along the x, y, and z dimensions was set as 75.26 × 48 x 66.56. These parameters were set to cover the entire 3-dimensional active sites of the protein. The output was saved in the grid parameter file (GPF) file format. The better the docking effect, the binding energy ≤−5.0 kJ/mol (1.207kcal/mol) is generally used as the evaluation criterion for molecular docking (35). Finally, molecular docking models and interactions were drawn by PyMOL 2.3.0 (36).

### 2.7 Analysis of immunomodulatory genetics, immune checkpoint genes, immunocytometrics, and immune infiltration

For immunomodulatory genetic analysis, the pan-cancer data-set The Cancer Genome Atlas (TCGA) TARGET Genotype-Tissue Expression (GTEx) (PANCAN; N=19,131; G=60,499) was downloaded from University of California Santa Cruz (UCSC; https://xenabrowser.net/) (37). The expression data for ADAM10 and 150 marker genes of five types of immune pathways [chemokine (38), acceptor (18), MHC (21), immunosuppressor (24), immunostimulator (39)] in each sample were extracted. The sample sources set as follows: Primary solid tumor, primary blood-derived cancer (peripheral blood), and primary blood-derived cancer (bone marrow). Samples were screened and normal were filtered. The samples with an expression level of 0 were also filtered, then each expression value was transformed by log2 (x+0.001). The Pearson correlations between ADAM10 and the markers of the five immune pathways were analyzed.

For analysis of immune checkpoint genes, the expression data for ADAM10 and 60 markers of 2 types of immune checkpoint pathways (stimulatory (36) and inhibitory (24)) in each sample were extracted in TCGA TARGET GTEx. The samples were the same from immunomodulatory genetic analysis with log2 (x+0.001). The Pearson correlation between ADAM10 and the marker genes of the two types of immune checkpoint was calculated.

For immunocytometric analysis, the expression data of ADAM10 in UCSC were obtained and the samples were screened and filtered as aforementioned. The expression data for each tumor were extracted and the expression profile was mapped to the GeneSymbol. The score of immune cell infiltration of each cancer patient according to gene expression was re-evaluated using the Tumor Immune Estimation Resource, deconvo_ips and deconvo_CIBERSOR methods (40–42) of the R package Immuno-Oncology Biological Research (version 0.99.9) (38). For immune infiltration analysis, the aforementioned data were processed after obtaining the ADAM10 expression data from UCSC. The R package ESTIMATE (version 1.0.13) (43) was applied to determine stromal, immune, and ESTIMATE scores for each tumor in each patient.

### 2.8 Statistical analysis

Data from more than three experimental repeats were obtained and analyzed using SPSS Statistics 25 (IBM Corp.). Comparison with a control group without drug intervention in each figure legend, and ordinary one‐way ANOVA or unpaired Student’s t tests were used for comparisons among groups.

## 3 Results

### 3.1 AD inhibits ADAM10 expression in various cancer cells

**Figure 1:**
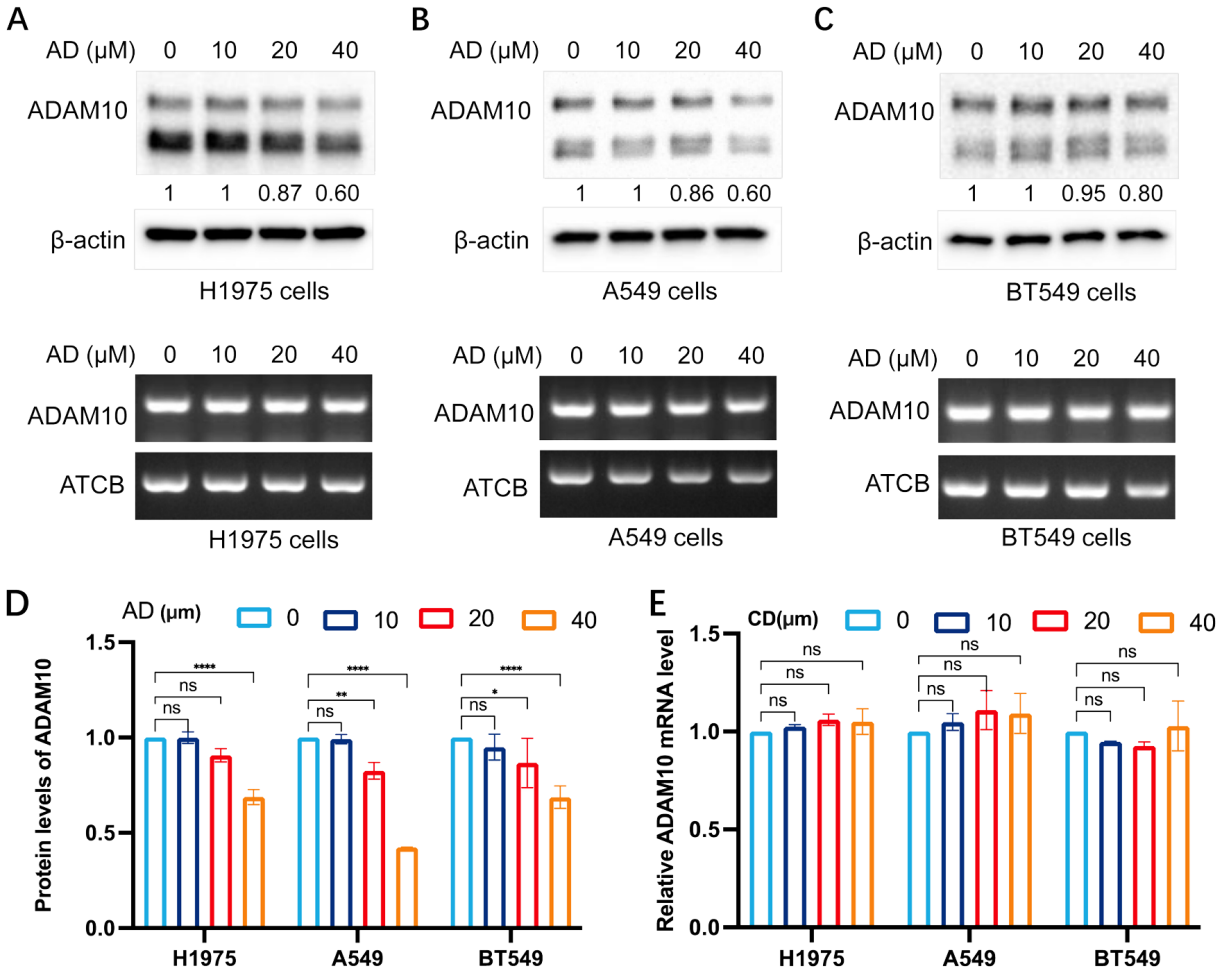
AD inhibits ADAM10 expression in various cancer cells. **(A)** AD inhibited expression of ADAM10 in both protein showing in upper panel and mRNA showing in lower panel in the H1975 lung cancer cells. **(B)** AD inhibited expression of ADAM10 in both protein showing in upper panel and mRNA showing in lower panel in the A549 lung cancer cells. **(C)** AD inhibited expression of ADAM10 in both protein showing in upper panel and mRNA showing in lower panel in the BT549 breast cancer cells. **(D)** Quantitative results for ADAM10 protein levels. **(E)** Quantitative results for ADAM10 mRNA levels. AD, adenosine; ADAM10, a disintegrin and metalloproteinase domain 10. *P <0.05; **P < 0.005; ****P < 0.0001.

Small molecules, such as AD, and CD and m^6^_2_A (both analogs of AD), may regulate ADAM10 expression, thus the expression regulation of ADAM10 in various cancer cells, mainly in lung cancer or breast cancer, was further investigated. AD at different concentrations was added, and western blotting and semi-quantitative RT-PCR were conducted. The results are shown in **Figure 1**. AD inhibited ADAM10 protein expression, but not mRNA expression in H1975 (**Figure 1A**) and A549 (**Figure 1B**) lung cancer cell lines, and BT549 breast cancer cell line (**Figure 1C**) in a dosage-dependent manner. Quantitative results for ADAM10 protein levels and quantitative results for ADAM10 mRNA levels are shown in **Figures 1D, E**, respectively.

### 3.2 CD inhibits ADAM10 expression in various cancer cell lines

**Figure 2:**
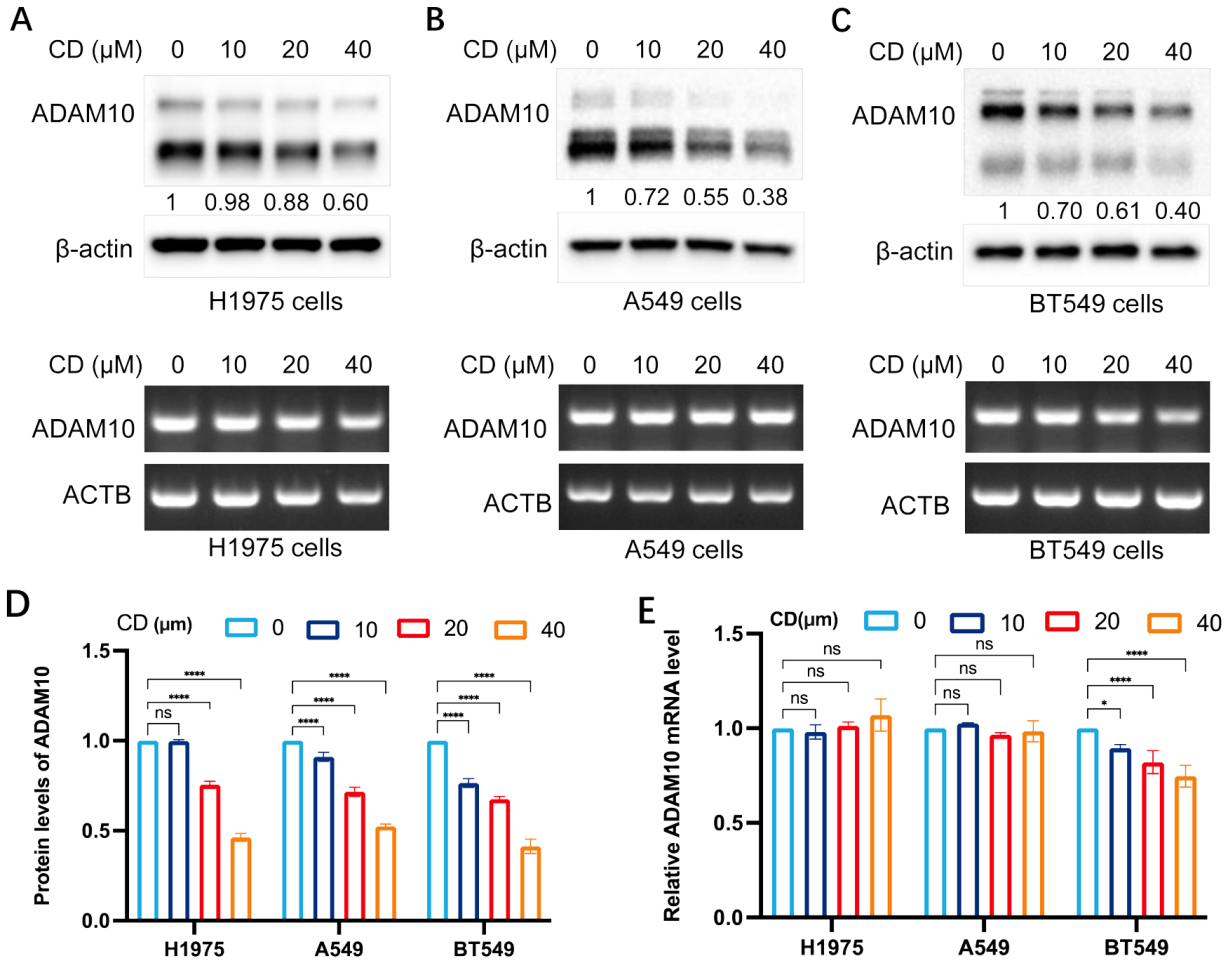
Cordycepin (CD) inhibits ADAM10 expressions in various cancer cells. **(A)** CD inhibited expression of ADAM10 in both protein showing in upper panel and mRNA showing in lower panel in the H1975 lung cancer cell line. **(B)** CD inhibited expression of ADAM10 in protein showing in upper panel and mRNA showing in lower panel in the A549 lung cancer cell line. **(C)** CD inhibited expression of ADAM10 in protein showing in upper panel and mRNA showing in lower panel in the BT549 breast cancer cell line. **(D)** Quantitative results for ADAM10 protein levels. **(E)** Quantitative results for ADAM10 mRNA levels. CD, cordycepin; ADAM10, a disintegrin and metalloproteinase domain 10. *P <0.05; ****P < 0.0001.

The present study subsequently investigated the expression regulation of ADAM10 by treatments with CD, an analog of AD. The results are shown in **Figure 2**. CD inhibited ADAM10 protein expression not mRNA expression in H1975 (**Figure 2A**) and A549 (**Figure 2B**) lung cancer cell lines, and the BT549 breast cancer cell line (**Figure 2C**), except mRNA expression in BT549 cells, in a dosage-dependent manner. Quantitative results for ADAM10 protein levels and Quantitative results for ADAM10 mRNA levels are shown in **Figures 2D, E**, respectively.

### 3.3 m^6^_2_A inhibits ADAM10 expression in various cancer cells

**Figure 3:**
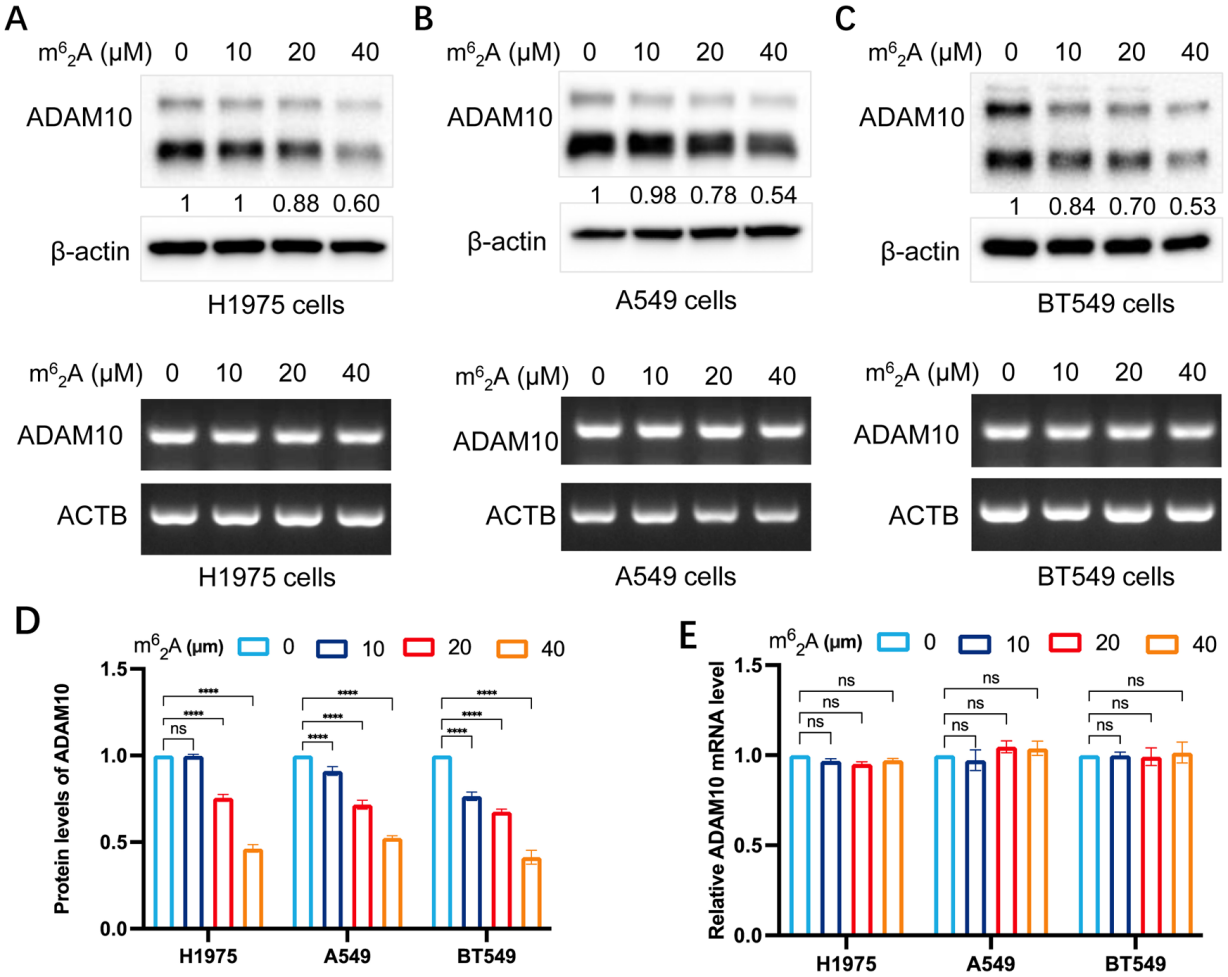
m^6^_2_A inhibits ADAM10 expressions in various cancer cell lines. **(A)** m^6^_2_A inhibited expression of ADAM10 in both protein showing in upper panel and mRNA showing in lower panel in the H1975 lung cancer cell line. **(B)** m^6^_2_A inhibited expression of ADAM10 in both protein showing in upper panel and mRNA showing in lower panel in the A549 lung cancer cell line. **(C)** m^6^_2_A inhibited expression of ADAM10 in both protein showing in upper panel and mRNA showing in lower panel in the BT549 breast cancer cell line. **(D)** Quantitative results for ADAM10 protein levels. **(E)** Quantitative results for ADAM10 mRNA levels. m^6^_2_A, N6, N6-dimethyladenosine; ADAM10, a disintegrin and metalloproteinase domain 10. ****P < 0.0001.

The present study also investigated the expression regulation of ADAM10 by treatment with m^6^_2_A, another analog of AD. The results are shown in **Figure 3**. m^6^_2_A inhibited ADAM10 protein expression but not mRNA expression in H1975 (**Figure 3A**) and A549 (**Figure 3B**) lung cancer cell lines, and BT549 breast cancer cell line (**Figure 3C**) in a dosage-dependent manner. Quantitative results for ADAM10 protein levels and quantitative results for ADAM10 mRNA levels are shown in **Figures 3D, E**, respectively.

### 3.4 ADAM10 stability by AD and CD

**Figure 4:**
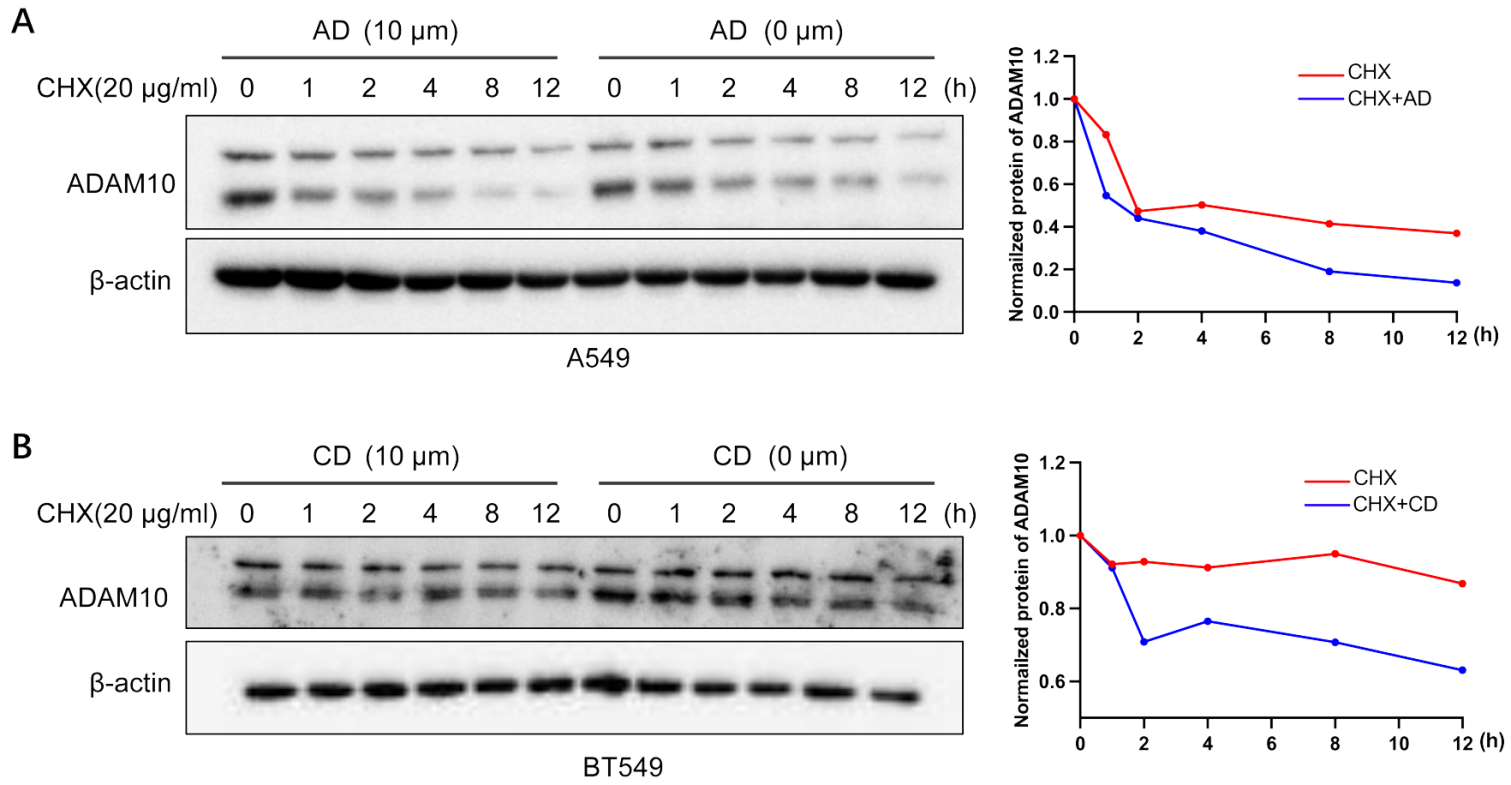
ADAM10 stability by AD and CD. **(A)** ADAM10 stability by AD treatments. Right panel, quantitative results. **(B)** ADAM10 stability by CD treatments. Right panel, quantitative results. CHX (20µg/ml) was used to treat A549 lung cancer cell lines or BT549 breast cancer cell lines with or without indicated AD or CD treatments, and western blot was performed. β-actin was used as an internal control. CHX, cycloheximide.

CHX is an inhibitor for protein synthesis. CHX chase assays are an experimental technique to monitor steady state protein stability. The protein stability of ADAM10 was demonstrated in A549 cells treated with CHX in the presence or absence of AD. In CHX-treated cell lines, the half-life of ADAM10 protein was >4 hours. However, the addition of AD significantly shorten the half-life of the protein of ADAM10 to less than 3 hours (**Figure 4A**). Furthermore, the combination of AD and CHX significantly promoted ADAM10 protein degradation rate by approximately 50% compared to CHX treatment alone (**Figure 4A**). Such results were also presented in BT549 cells by addition of CD or CHX alone and their combination (**Figure 4B**). Altogether, our results suggest that AD or CD treatment may not inhibit ADAM10 translation but promotes the degradation.

### 3.5 Molecular docking for small molecules model diagram

**Figure 5:**
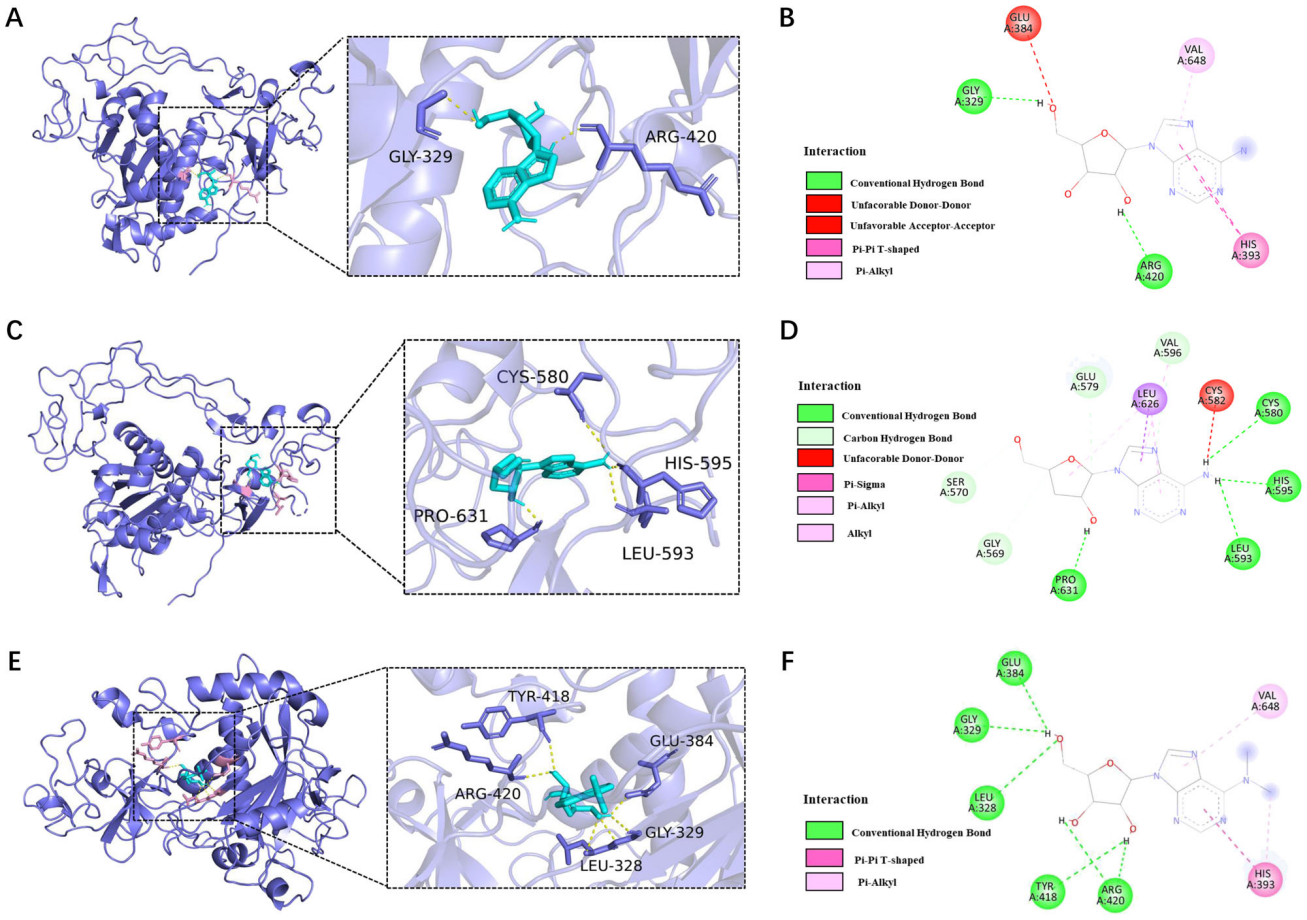
Molecular docking for ADAM10 (6bdz) and AD, CD, and m^6^_2_A binding sites. **(A)** Molecular docking of AD with human ADAM10 protein (6bdz) and active site residues and **(B)** force of attractions involved in docking; **(C)** Molecular docking of CD with human ADAM10 protein (6bdz) and active site residues and **(D)** force of attractions involved in docking; **(E)** Molecular docking of AD with human ADAM10 protein (6bdz) and active site residues and **(F)** force of attractions involved in docking.

Molecular docking of AD, CD, and m^6^_2_A with ADAM10 was performed. For the molecular docking results, the lower the binding energy, the higher the possibility of interaction when the conformation of the ligand-receptor binding is stabilized at binding energy <0 kJ/mol, the good affinity at binding energy ≤-5.0 kJ/mol (-1.207 kcal/mol), and the very good affinity at binding energy ≤-7.0 kJ/mol (-1.673 kcal/mol). The docking results of this study are shown in **Table 1** and the results are visualized in **Figure 5**.

**Table 1:** ADAM10 (6bdz) molecular docking binding sites and energy.

Molecular name	Binding sites	Binding Energy (Kcal/mol)
Adenosine(AD)	GLY-329、ARG-420	-7.104
Cordycepin(CD)	CYS-580、LEU-593、HIS-595、PRO-631	-7.609
m^6^_2_A	LEU-328、 GLY-329、GLU-384、TYR-418, ARG-420	-7.215

Avery good affinity if the binding energy is ≤ -7.0 kJ/mol (1.673 kcal/mol), and the smaller the free binding energy, the higher the binding potential. 1kcal/mol=4.184kj/mol.

The binding energy of AD to ADAM10 protein was -7.104kcal/mol, and there were 2 binding residues, namely GLY-329 and ARG-420, indicating that adenosine can well match the active pocket of ADAM10, AD Glycoside is a potential ADAM10 target inhibitor (**Figures 5A, B**).

The binding energy of CD to ADAM10 protein is -7.609kcal/mol. Potential binding sites included amino acid residues such as CYS-580, LEU-593, HIS-595, PRO-631, which can form strong hydrogen bonds. Plays an important role in stabilizing small molecule compounds (**Figures 5C, D**).

The binding energy of m^6^_2_A to ADAM10 protein was -7.215 kcal/mol, and the potential binding sites were LEU-328, GLY-329, GLU-384, TYR-418 and ARG-420, indicated that m^6^_2_A has a very good affinity with ADAM10 and had a stable conformation (**Figures 5E, F**).

### 3.6 ADAM10 expression is associated with immunoregulation in pan-cancers

**Figure 6:**
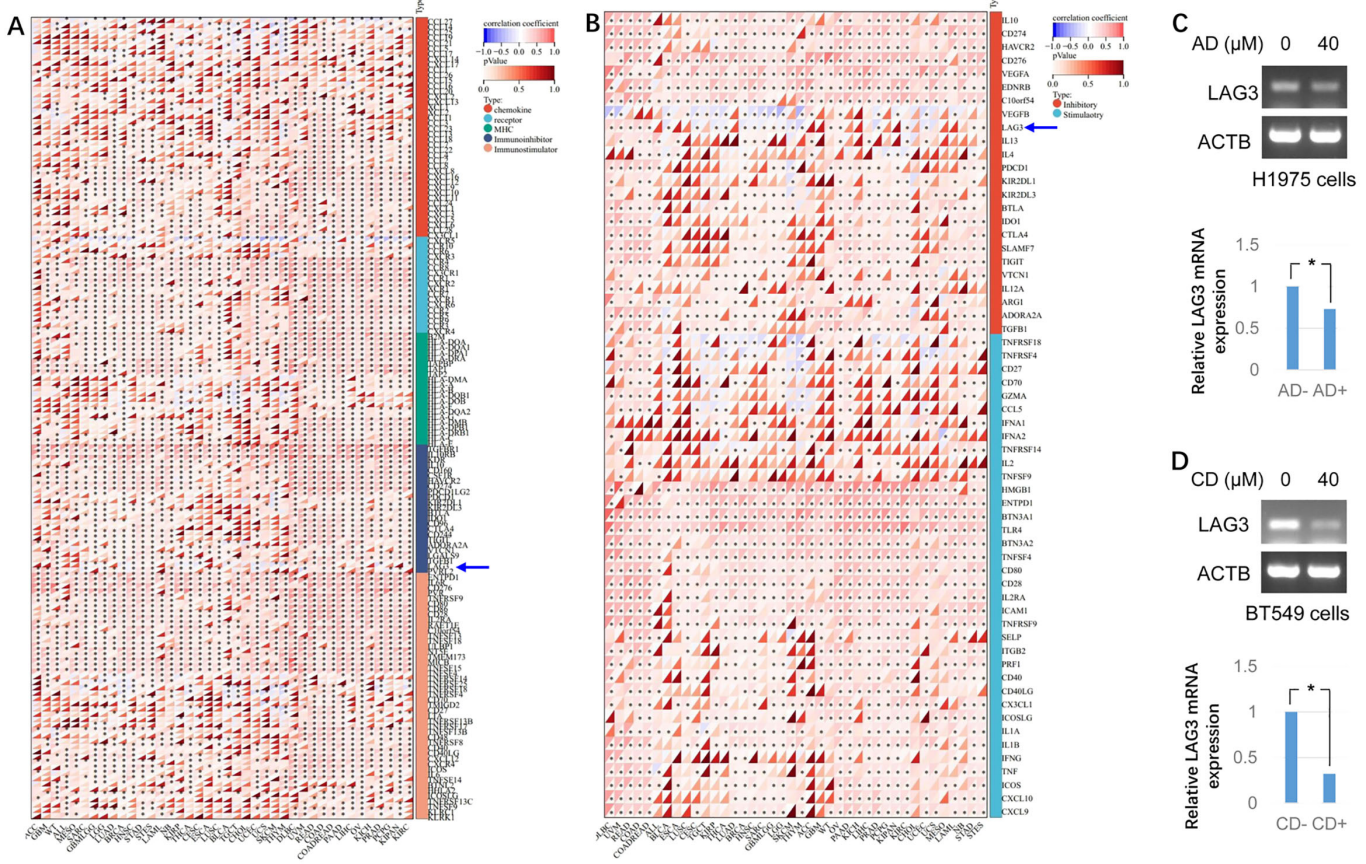
Bioinformatic analysis for the immunoregulatory actions of ADAM10 in various cancers. **(A)** Correlations between ADAM10 and 150 genes of Immunoregulation. There are 41 chemokines, 18 receptors, 21 MHCs, 24 immunoinhibitors, and 46 immunostimulators. **(B)** Correlations between ADAM10 and 60 genes of immune checkpoint pathways. **(C)** AD inhibits LAG3 expression in H1975 cells. Up panel indicates semi-quantitative RT-PCR, bottom panel indicates the quantitative results from up panel. **(D)** CD inhibits LAG3 expression in BT549 cells. Up panel indicates semi-quantitative RT-PCR, bottom panel indicates the quantitative results from up panel. *P <0.05. ADAM10, a disintegrin and metalloproteinase domain 10. For immunomodulatory genetic analysis, the pan-cancer data-set The Cancer Genome Atlas (TCGA) TARGET Genotype-Tissue Expression (GTEx) (PANCAN) was downloaded from University of California Santa Cruz (UCSC; https://xenabrowser.net/).

The present study further aimed to determine whether ADAM10 expression is correlated with immunoregulation in pan-cancers. It was revealed that ADAM10 expression was negatively associated with numerous immune regulatory genes, such as C-C motif chemokine ligand (CCL)27, CCL14, CCL25, C-X-C motif chemokine receptor 5 (CXCR5), HLA-B, HLA-DOB1, LAG3, TNF receptor superfamily member 18 (TNFRSF18), and TNF receptor superfamily member 4 (TNFRSF4) in bladder urothelial carcinoma (BLCA), thymoma (THYM), breast invasive carcinoma (BRCA), TGCT, kidney renal papillary cell carcinoma (KIRP), SKCM and thyroid carcinoma (THCA) (**Figure 6A**). Moreover, the expression of ADAM10 was reciprocally exclusive with some tumor immune checkpoint genes, such as VEGFB, LAG3, IL4, TNFRSF18, TNFRSF4, TNF receptor superfamily member 14 (TNFRSF14), CD27, CCL5, etc. (**Figure 6B**). Since ADAM10 expression was negatively associated with LAG3 (**Figures 6A, B**, blue arrows) and it was reported that ADAM10 regulates constitutive LAG3 cleavage (44), further validation need to perform. To validate the aforementioned bioinformatics results, semi-quantitative-RT-PCR was conducted and the results revealed that *LAG3* mRNA levels were reduced by both AD and CD treatment (**Figures 6C, D**) in cancer cells.

**Figure 7:**
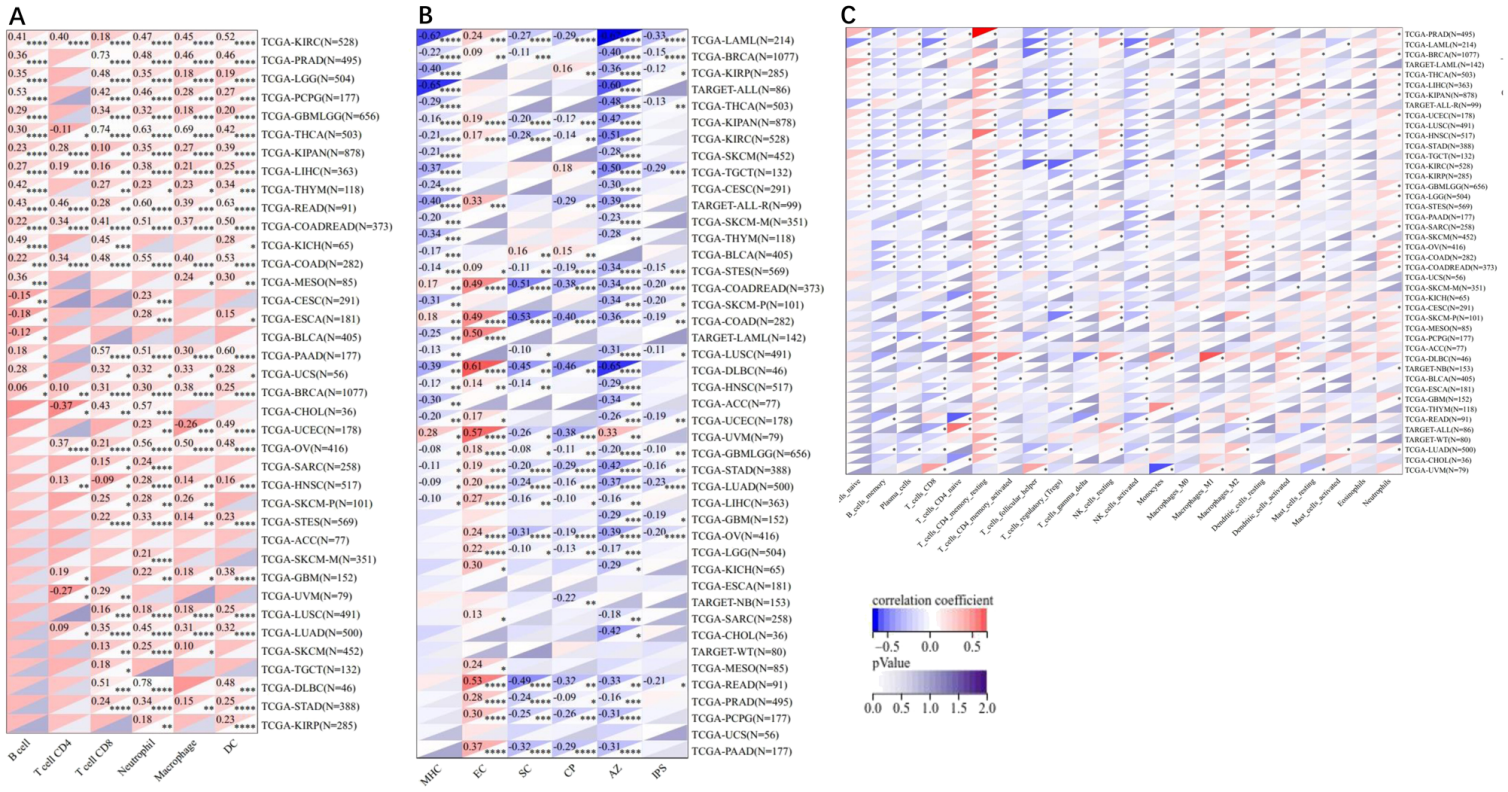
Pearson’s correlations of the correlation between ADAM10 expression and tumor-related immune cells in various cancers calculated using different methods. **(A)** Correlation between ADAM10 and six tumor-associated immune cells calculated using Tumor Immune Estimation Resource. **(B)** Correlations between ADAM10 and six tumor-related immune cells calculated using deconvo_ips. **(C)** Correlations between ADAM10 and 22 tumor-associated immune cells calculated using deconvo CIBERSOR. *P <0.05; **P < 0.005; ***P <0.001; ****P < 0.0001. ADAM10, a disintegrin and metalloproteinase domain 10.

The infiltration scores of 6 immune cells including lymphocyte B, lymphocyte T CD4, macrophage, lymphocyte T CD8, neutrophil, and dendritic cells were reappraised for 9,405 tumor samples in 38 cancer types, 6 immune cells including SC, MHC, endothelial cell, induced pluripotent stem cells, CP and AZ, and 22 class immunocytes in 10,179 tumor specimens from 44 cancers based on the *ADAM10* gene expression. The results demonstrated that the *ADAM10* expression was associated with immune infiltration in 34 tumor species, including TCGA-BLCA, TCGA-BRCA, TCGA-cervical squamous cell carcinoma and endocervical adenocarcinoma, TCGA-CHOL, TCGA-COAD, TCGA-READ, TCGA-lymphoid neoplasm diffuse large B-cell lymphoma, TCGA-GBM, TCGA-LGG, TCGA-head and neck squamous cell carcinoma (HNSC), TCGA-kidney chromophobe, TCGA-KIPAN, TCGA-KIRC, TCGA-KIRP, TCGA-LGG, TCGA-liver hepatocellular carcinoma, TCGA-lung adenocarcinoma (LUAD), TCGA-LUSC, TCGA-ovarian serous cystadenocarcinoma (OV), TCGA-PAAD, TCGA-pheochromocytoma and paraganglioma, TCGA-PRAD, TCGA-READ, TCGA-sarcoma (SARC), TCGA-SKCM-M, TCGA-SKCM-P, TCGA-SKCM, TCGA-STAD, TCGA- stomach and esophageal carcinoma (STES), TCGA-TGCT, TCGA-THCA, TCGA-uterine corpus endometrial carcinoma, TCGA-THYM and TCGA-uveal melanoma (**Figures 7A–C**).

**Figure 8:**
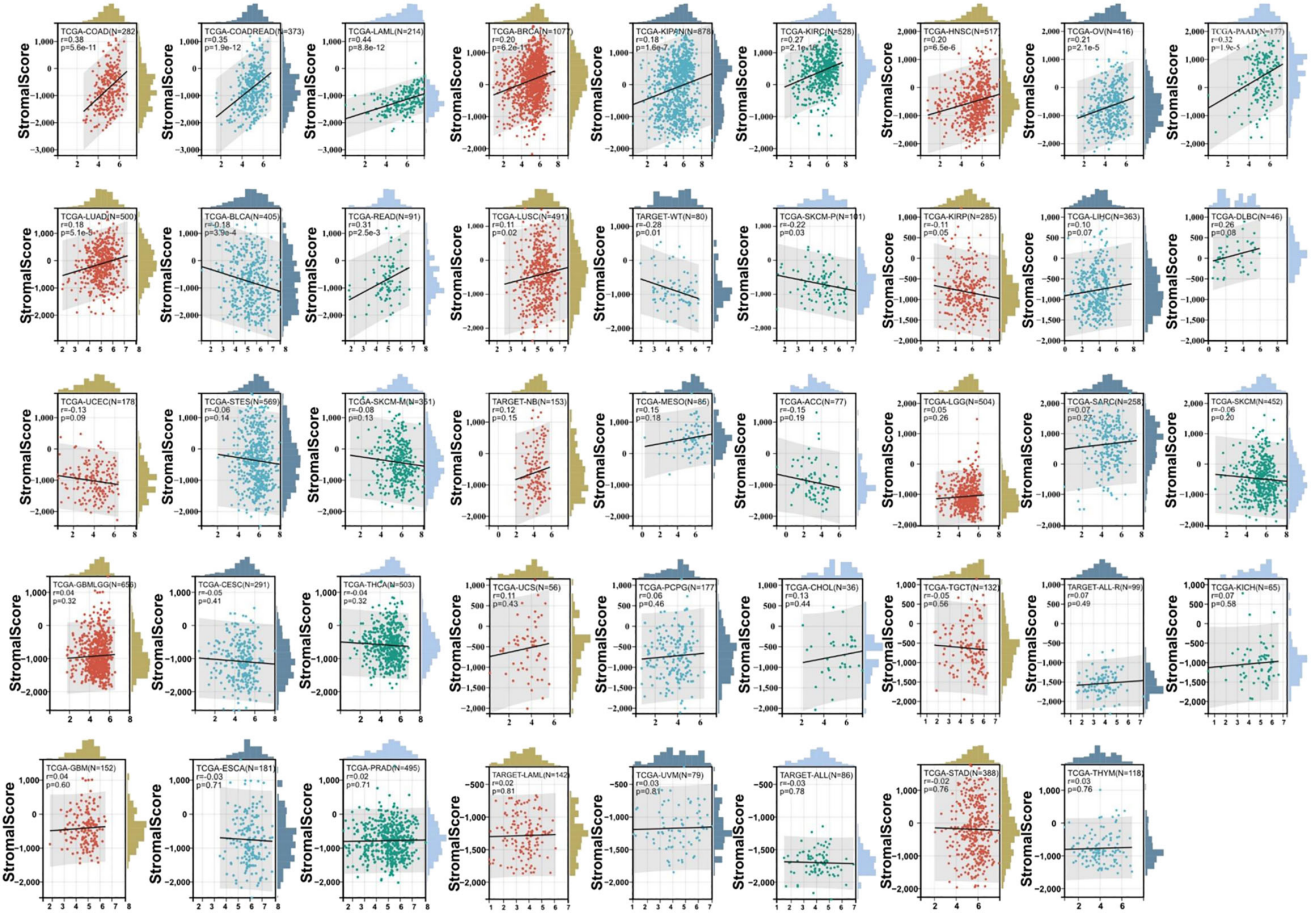
Association between ADAM10 expression and immune infiltration score in multiple types of cancer. For immune infiltration analysis, the pan-cancer data-set TCGA TARGET Genotype-Tissue Expression (GTEx) was also downloaded from UCSC. ADAM10 expression and immune infiltration score in multiple types of cancer are presented in the indicated different panels.

The association between the score of immune invasion and ADAM10 expression in cancer was investigated. The *ADAM10* expression was associated with immune invasion in 15 neoplasm species according to the stromal score, indicating 12 significant positive associations (TCGA-BRCA, TCGA-LUAD, TCGA-KIPAN, TCGA-COAD, CGA-COADREAD, TCGA-HNSC, TCGA-KIRC, TCGA-LUSC, TCGA-READ, TCGA-OV, TCGA-PAAD, and TCGA-acute myeloid leukemia) and three significant negative associations (TARGET-WT, TCGA-SKCM-P and TCGA-BLCA) when analyzing the association between ADAM10 and immune infiltration markers in 10,179 specimens from 44 cancer types (**Figure 8**; **Table 2**).

**Table 2:** Immuno-infiltration analysis of ADAM10 gene in pan-cancer.

Immu	StromalScore	StromalScore	ImmuneScore	ImmuneScore	ESTIMATEScore	ESTIMATEScore
Method	pearson_R	pearson_P	pearson_R	pearson_P	pearson_R	pearson_P
TCGA-GBM(N=152)	0.04271416	0.60131741	-0.012240564	0.881023685	0.01161644	0.88704869
TCGA-GBMLGG(N=656)	0.03895496	0.31914856	-0.025938873	0.507199996	0.00046776	0.99045934
TCGA-LGG(N=504)	0.05066056	0.25627817	-0.029929049	0.502610912	0.00099452	0.98223142
TCGA-UCEC(N=178)	-0.1291504	0.08577021	-0.264091369	0.000367976	-0.2317808	0.00185158
TARGET-LAML(N=142)	0.02063634	0.80741451	0.042451999	0.615928569	0.03541143	0.67567118
TCGA-BRCA(N=1077)	0.19754147	6.1572E-11	-0.089211382	0.003387933	0.0432791	0.15580129
TCGA-CESC(N=291)	-0.0485548	0.40925514	-0.210414907	0.000300917	-0.1597527	0.00631408
TCGA-LUAD(N=500)	0.18006644	5.1332E-05	0.017771814	0.691790397	0.09946051	0.02615146
TCGA-ESCA(N=181)	-0.0280536	0.70774837	-0.157348704	0.034392475	-0.101483	0.17403011
TCGA-STES(N=569)	-0.0611938	0.14487988	-0.081600932	0.051720751	-0.0773408	0.06524564
TCGA-SARC(N=258)	0.06915533	0.26841034	-0.05194145	0.406079279	-0.0050576	0.93556671
TCGA-KIRP(N=285)	-0.1145228	0.05345483	-0.298189027	2.91221E-07	-0.238228	4.8504E-05
TCGA-KIPAN(N=878)	0.17567384	1.6164E-07	-0.001085985	0.974365937	0.08684342	0.0100396
TCGA-COAD(N=282)	0.37737003	5.6451E-11	0.406562737	1.19162E-12	0.41355754	4.4639E-13
TCGA-COADREAD(N=373)	0.35401574	1.873E-12	0.375026269	6.68498E-14	0.38495776	1.2689E-14
TCGA-PRAD(N=495)	0.01659088	0.71271229	-0.098322402	0.028720116	-0.0511213	0.25627325
TCGA-STAD(N=388)	-0.0154993	0.76087357	0.005842502	0.908672118	-0.0051012	0.92021812
TCGA-HNSC(N=517)	0.19678791	6.5499E-06	-0.0527557	0.231123287	0.07335364	0.09569423
TCGA-KIRC(N=528)	0.27185927	2.1244E-10	0.000387726	0.99290838	0.13074072	0.00261246
TCGA-LUSC(N=491)	0.10704371	0.01765835	-0.078789679	0.081137303	0.00865911	0.84822196
TCGA-THYM(N=118)	0.0288897	0.75614533	-0.251465419	0.006017624	-0.1714422	0.06341332
TCGA-LIHC(N=363)	0.0953168	0.06969354	-0.097169073	0.064412685	-0.0166012	0.7525897
TARGET-WT(N=80)	-0.2767923	0.01293663	-0.377626362	0.000553911	-0.3561183	0.00118662
TCGA-SKCM-P(N=101)	-0.2177879	0.02868193	-0.348432101	0.000356041	-0.3272199	0.00083768
TCGA-SKCM(N=452)	-0.0604256	0.19974436	-0.136509667	0.003639547	-0.1165019	0.01319541
TCGA-BLCA(N=405)	-0.1754626	0.00038851	-0.235744315	1.60754E-06	-0.2196155	8.1735E-06
TCGA-SKCM-M(N=351)	-0.0815595	0.12723232	-0.157710522	0.003049254	-0.1393309	0.00895381
TCGA-THCA(N=503)	-0.0443567	0.3207929	-0.197902975	7.7578E-06	-0.148677	0.00082345
TARGET-NB(N=153)	0.11732753	0.14863961	0.099604274	0.220582052	0.11643718	0.15176719
TCGA-MESO(N=85)	0.14544754	0.18411851	-0.023784521	0.828936133	0.05148679	0.63980821
TCGA-READ(N=91)	0.313106	0.00251175	0.287678537	0.005691405	0.3187141	0.00207678
TCGA-OV(N=416)	0.20690006	2.1058E-05	0.073150937	0.136356021	0.14621595	0.00279581
TCGA-UVM(N=79)	0.0280013	0.80648584	0.142644479	0.209817475	0.11261228	0.32309709
TCGA-PAAD(N=177)	0.31564478	1.8727E-05	0.141225445	0.060795689	0.23922154	0.00134231
TCGA-TGCT(N=132)	-0.0505709	0.56471112	-0.282583853	0.00102731	-0.2466561	0.00435686
TCGA-UCS(N=56)	0.10861283	0.42555935	-0.063505395	0.641944238	0.01798445	0.89533354
TCGA-LAML(N=214)	0.44455444	8.8358E-12	0.063189963	0.357627566	0.25292101	0.00018459
TARGET-ALL(N=86) TCGA-PCPG(N=177)	-0.0304825 0.05543334	0.78054296 0.46366144	-0.160995345 -0.073613193	0.138649026 0.330184297	-0.1318742 -0.0085451	0.22614477 0.91012461
TCGA-ACC(N=77)	-0.1504396	0.19156722	-0.273077659	0.016264935	-0.2311589	0.04310219
TARGET-ALL-R(N=99)	0.07033647	0.48905761	0.093281292	0.358433559	0.09233245	0.36336475
TCGA-DLBC(N=46)	0.26240476	0.07811087	0.477704056	0.000787103	0.44587593	0.00189918
TCGA-KICH(N=65)	0.07059839	0.57627334	-0.039322809	0.75580099	0.00888969	0.94396949
TCGA-CHOL(N=36)	0.13183983	0.44339272	-0.071474366	0.678696896	0.00535474	0.97527374

The aforementioned data suggested that ADAM10 was negatively associated with tumor immunosuppression and interrelated with the immune infiltration of tumors, and ADAM10 might be an important molecular target in patients with cancer.

## 4 Discussion

AD is an endogenous purine nucleoside that is formed of an adenine moiety connected to a ribose sugar molecule moiety and is found in all organs, tissues and cells. Accumulating evidence has revealed that AD promotes apoptosis in a variety of cancer cells (45). CD is an AD analog showing various pharmacological effects and may confer resistance to various tumors (22, 46), and viruses (23, 39), such as SARS-CoV-2 (47, 48). A previous study identified host ADAM17, a metalloprotease, as a facilitator of SARS-CoV-2 cell invasion and the ADAM10, another metalloprotease, as a co-factor required for lung cell syncytia formation (16). Based on our previous research m^6^_2_A is functionally related to AD, which exhibits an inhibitory effect on ADAM17 protein expression (49). Furthermore, in this study, AD and its analogs, CD and m^6^_2_A, inhibit ADAM10 expression in various cancer cells, indicating their anti-cancer potential.

Molecular docking is based on the prediction and evaluation of small-molecules at the molecular level. The interaction with target proteins, the binding site of the analyzed and the binding affinity of the two, complement the predictive power of network pharmacology in terms of molecular structure. In our study, molecular docking was carried out (50). In the docking simulation phase, the AutoDock Vina program explores the conformational state of the docked small-molecules based on a conformational search algorithm and evaluates the ligand-receptor interactions at each point of the docking simulation based on an energy grid generated by AutoGrid. We performed molecular docking of small-molecules (AD, CD and m^6^_2_A), with ADAM10 protein and found the binding energies of all docking groups were <-7cal/mol, suggesting that the binding activities were very well. Thus, our docking provides a potential basis for explaining the role of drug/small molecules on ADAM10 protein at the sub-level and suggests new possibilities for potential small-molecules that have not yet been focused on in basic research on ADAM10. Our future will screen more small molecules by molecular docking simulations and/or experimental validation for inhibiting ADAM10 expression and investigating the resulting mechanism, such as DNA/RNA G-quadruplex modification, that may develop a potential therapeutic strategy for anti-cancer.

The immunomodulatory role of ADAM10 expression in cancer was subsequently investigated. Bioinformatics analysis revealed that ADAM10 was negatively associated with immunomodulatory genes such as CCL27, CCL14, CCL25, CXCR5, HLA-B, HLA-DOB1, LAG3, TNFRSF18, and TNFRSF4 in BLCA, THYM, BRCA, TGCT, KIRP, SKCM, and THCA, suggesting the immune promoting role of ADAM10. ADAM10 was mutually exclusive with numerous tumor immune checkpoints, including EGFB, LAG3, IL4, TNFRSF18, TNFRSF4, TNFRSF14, CD27 and CCL5, which further suggests that ADAM10 may be a novel target for tumor immunotherapy with good potential. Among the aforementioned genes, CCL14 can enhance the activation of immune cells, and the expression of CCL14 in cancer is associated with prognosis. CCL14 also exhibits an association with CD8 T cells, B cells, CD4 cells and macrophage cells (51). CXCR5 is a receptor of CXCL13, and these form a CXCL13-CXCR5 signaling axis to regulate inflammatory diseases and tumors (52).

LAG3 is a cell surface inhibitory receptor that co-localizes with CD3, CD4 and CD8 molecules within lipid rafts. LAG3 exhibits multiple activities for T-cell activation and effector functions (53), and serves roles as an inhibitory immune checkpoint molecule in immunity. Besides programmed cell death protein 1 (PD-1) and cytotoxic T-lymphocyte associated protein 4, LAG3 is the third inhibitory receptor as a target for promoting anti-cancer responses and immunotherapies. Similar to PD-1, constitutive LAG3 expression is associated with exhausted T cells (CD4 and CD8 T cells) in cancer and chronic viral invasions [14–19]. LAG3 can be translocated away from the cell membrane by metalloproteinases ADAM10 and ADAM17 to exert its functions (54). Specifically, ADAM10 regulates constitutive LAG3 cleavage, while ADAM17 regulates LAG3 cleavage induced by T cell receptor signaling (44), where ADAM17 showed immune and SARS-CoV-2 entry for the expression in patients with cancer (49). As the downstream signaling of ADAM10, the effect of AD and CD on LAG3 expression was further examined in the present study. Both AD and CD inhibited LAG3 expression *in vitro*, indicating the possible pathways of AD/ADAM10/LAG3 or CD/ADAM10/LAG3 in anti-cancers, or suggesting a potential method for immunotherapy of cancers. However, there are the challenges of this approach/targeting. For example, the potential off-target effects of ADAM10 inhibition, the need for further preclinical and clinical studies, and the potential for combination therapies with other immunomodulatory agents, etc. All these studies may be need to be conducted in the future.

The present study further revealed that ADAM10 was negatively associated with T cells CD4, T cells CD4 naive, macrophages, MHCs, AZs, monocytes, and other immune cells associated with tumor infiltration. The tumor microenvironment (TME) is composed of fibroblasts, and vascular endothelial and immune cells, and it affects the occurrence and development of cancer by interacting with cells around the circulatory system and lymphatic system (55). Immune cells are a vital component of the tumor matrix, and serve a critical role in tumor maintenance and development. Evidence has shown that innate immune cells, such as macrophages, neutrophils, dendritic cells and natural killer cells, and adaptive immune cells, such as T cells and B cells, exist in the TME and promote tumor progression (56). Tumor-associated macrophages (TAMs) are generally divided into two functional subtypes, including classically activated macrophages M1 and M2. Evidence shows that the degree of TAM infiltration in the tumor is related to the cancer outcome (57). Liang et al. (58) suggested that regulatory T cells, M2 macrophages and other immune cells promote tumor growth in the TME. The former has anti-cancer functions, including regulating cytotoxicity and killing tumor cells; the latter could promote the occurrence and metastasis of cancer cells, inhibit the anti-cancer effects mediated by T cells, and lead to cancer deterioration. M1 and M2 macrophages have high plasticity, so they can be transformed into each other to change the TME or intervene in therapy (59). Thus, ADAM10 expression may regulate the homeostasis of the TME by regulating a variety of immune cells and immune regulatory genes. However, there are limitations for the gene immune regulation in cancer, such as the lack of *in vivo*/*in vitro* validation.

## 5 Conclusion

The present study indicated ADAM10 expression regulation by AD, CD and m^6^_2_A, and in AD/ADAM10/LAG3 or CD/ADAM10/LAG3 signaling in cancers, suggesting a potential method for immunotherapy of cancers by targeting ADAM10 using the small molecules AD, CD and m^6^_2_A.

## Data Availability

The original contributions presented in the study are included in the article/supplementary material. Further inquiries can be directed to the corresponding authors.
