# Corrigendum: A disintegrin and metalloproteinase domain 10 expression inhibition by the small molecules adenosine, cordycepin and N6, N6-dimethyladenosine and immune regulation in malignant cancers

**DOI:** 10.3389/fimmu.2024.1518823

**Published:** 2024-11-13

**Authors:** Wenqian Zhang, Jiewen Fu, Jiaman Du, Xiaoyan Liu, Jingliang Cheng, Chunli Wei, Youhua Xu, Junjiang Fu

**Affiliations:** ^1^ Key Laboratory of Epigenetics and Oncology, The Research Center for Preclinical Medicine, Southwest Medical University, Luzhou, Sichuan, China; ^2^ Department of Rehabilitation Medicine, The Affiliated Hospital of Southwest Medical University, Luzhou, Sichuan, China; ^3^ State Key Laboratory of Quality Research in Chinese Medicine, Faculty of Chinese Medicine, Macau University of Science and Technology, Taipa, Macau SAR, China

**Keywords:** ADAM10, expression, pan-cancer, immunotherapy, adenosine, CD, m^6^_2_A

In the published article, there was an error in affiliation(s) Youhua Xu^1,*^. Instead of “Youhua Xu ^1,*^”, it should be “Youhua Xu ^3,*^”.

The authors apologize for this error and state that this does not change the scientific conclusions of the article in any way. The original article has been updated.

